# Multi-omics analysis of the Indian ovarian cancer cohort revealed histotype-specific mutation and gene expression patterns

**DOI:** 10.3389/fgene.2023.1102114

**Published:** 2023-04-06

**Authors:** Anisha Mhatre, Jinsha Koroth, Meghana Manjunath, Sandeep Kumar S, Ramesh Gawari, Bibha Choudhary

**Affiliations:** ^1^ Department of Biotechnology and Bioinformatics, Institute of Bioinformatics and Applied Biotechnology, Bangalore, Karnataka, India; ^2^ Graduate Student Registered Under Manipal Academy of Higher Education, Manipal, Karnataka, India; ^3^ Kidwai Cancer Institute of Oncology, Bangalore, India

**Keywords:** OVCa, RNA-seq, exome, alternative splicing, miRNA

## Abstract

**Introduction:** In India, OVCa is women’s third most common and lethal cancer type, accounting for 6.7% of observed cancer incidences. The contribution of somatic mutations, aberrant expression of gene and splice forms in determining the cell fate, gene networks, tumour-specific variants, and the role of immune fraction infiltration have been proven essential in understanding tumorigenesis. However, their interplay in OVCa in a histotype-specific manner remains unclear in the Indian context. In the present study, we aimed to unravel the Indian population histotype-specific exome variants, differentially expressed gene modules, splice events and immune profiles of OVCa samples.

**Methods:** We analysed 10 tumour samples across 4 ovarian cancer histotypes along with 2 normal patient samples. This included BCFtool utilities and CNVkit for exome, WGCNA and DESeq2 for obtaining differential module hub genes and dysregulated miRNA targets, CIBERSORTx for individual immune profiles and rMATS for tumour specific splice variants.

**Result:** We identified population-specific novel mutations in Cancer Gene Census Tier1 and Tier2 genes. MUC16, MUC4, CIITA, and NCOR2 were among the most mutated genes, along with TP53. Transcriptome analysis showed significant overexpression of mutated genes MUC16, MUC4, and CIITA, whereas NCOR2 was downregulated. WGCNA revealed histotype-specific gene hubs and networks. Among the significant pathways, alteration in the immune system was one of the pathways, and immune profiling using CIBERSORTx revealed histotype-specific immune cell fraction. miRNA analysis revealed miR-200 family, miR-200a and miR-429 were upregulated in HGSOCs.Splice factor abrasion caused splicing perturbations, with the most abundant alternative splice event being exon skipping and the most spliced gene, SNHG17. Pathway analysis of spliced genes revealed translational elongation and Base excision repair as the pathways altered in OVCa.

**Conclusion:** Integrated exome, transcriptome, and splicing patterns revealed different population-specific molecular signatures of ovarian cancer histotypes in the Indian Cohort.

## 1 Introduction

Amongst gynecologic cancers, ovarian cancer has the worst prognosis, with approximately 3,14,000 new cases and 2,07,000 deaths a year (Cabasag et al., 2022), characterised by heterogeneity and distinct histotypes, unique molecular features, clinical features, and prognosis (Soslow, 2008) (Peres et al., 2018). According to the WHO classification, 90% of ovarian tumours arise from the epithelium, 3% from germ cells, and 2% from sex-cord stromal (Hanby and Walker, 2004) (Herrington and Simon Herrington, 2020). Whereas based on histotype-specific immune profile and molecular signatures, ovarian cancers are classified as high-grade serous carcinoma (HGSOC), low-grade serous carcinoma (LGSOC), endometrioid carcinoma (EC), clear cell carcinoma (CCC), and mucinous carcinoma (MC) (Kurman and Centre international de recherche sur le cancer, 2014) (W. H. O. Classification WHO Classification of Tumours Editorial Board and Who Classification of Tumours Editorial, 2020). Of all the cases, about 68% of ovarian cancers are High-grade serous ovarian cancers (Kurman and Shih, 2016), whereas 5% of them are LGSCs, EC 10%, 6%–10% CCC, and 3%–4% MC (Prat et al., 2018). Cases are usually diagnosed in the later stages III and IV, when the tumour has spread beyond the abdomen, the diagnosis majorly relies on imaging techniques, CA-125 blood tests, and surgical biopsies, and is associated with poor prognosis (Roett and Evans, 2009). The 5-year survival of OVCa patients in India is about 45%.

The advent of Next-Generation Sequencing (NGS) has led to a better understanding of mechanistic insights driving different histotypes of ovarian cancer (Network and The Cancer Genome Atlas Research Network, 2011) (Cheasley et al., 2021). The Multi-Omics approach, the mutational analysis combined with gene expression, and regulation of gene expression *via* DNA methylation and miRNA have shed light on the key genomic alterations leading to the acquisition of hallmarks of cancer: resisting cell death and causing genomic instability, such as 96% of the cases with TP53 mutation, 22% having BRCA1 and BRCA2 mutations, CCNE1 amplification, and promoter methylation of 168 genes in HGSOC (Hanahan and Weinberg, 2011) (Wu et al., 2019). Mutations and epigenetic modifications in BRCA1 and BRCA2 have been reported in ∼50% of HGSOCs leading to Homologous recombination deficiency (HRD) (Konecny et al., 2014). In addition, mutations in HR genes ATM, BARD1, BRIP1, CHEK1, CHEK2, FAM175A, MRE11A, NBN, PALB2, RAD51C, and RAD51D have also been reported (Pennington et al., 2014).

Further, HGSOCs have been classified based on gene expression signatures as “immunoreactive,” “proliferative,” “differentiated,” and “mesenchymal” (Konecny et al., 2014). Apart from the TCGA study, genomic studies in LGSOC have identified ovarian cancers, with 47% of cases with mutations in the oncogenic RAS gene (KRAS, BRAF, and NRAS) and novel drivers such as USP9X (27%), MACF1 (11%), ARID1A (9%), NF2 (4%), DOT1L (6%), and ASH1L (4%) (Cheasley et al., 2021). Endometrioid tumours are characterised by *ß*-catenin alterations, microsatellite instability, and PTEN and POLE mutations. In contrast, ARID1A and PIK3CA mutations are associated with endometrioid and clear cell carcinomas. Mucinous carcinomas are uncommon tumours associated with copy-number loss of CDKN2A and KRAS alterations (Hollis et al., 2020) (Yamamoto et al., 2011) (Leo et al., 2021). The histological subtype and its diverse molecular features can be used for individualised clinical decision-making, avoiding toxicity due to therapy.

Despite advances in molecular pathology and targeted therapy, chemoresistance and relapse have led to poor ovarian cancer survival due to a lack of consideration for population and individual heterogeneity. In addition, methods of gene regulation such as alternative splicing and miRNA in ovarian cancer pathogenesis, have been established. Gene expression is further diversified by the alternative splicing (AS) of precursor mRNA, occurring in 95% of human exons. Thus its dysregulation has strongly implicated in cancer and about 30% more AS events are observed in malignant tumours than in normal tissues. The main causes of this dysregulation are splice factor mutations and their aberrant expression.

The cell surface glycoprotein CD44 isoform CD44v activates cell signaling pathways that induce Cancer Stem Cell (CSC) state (Zhang et al., 2019). Amongst several factors, CD44 splicing is regulated by splice factors, namely, ESRP1 and U2AF2 (Yae et al., 2012; Zhang et al., 2016). Also, splice factors UBAP2L, which regulates the expression of TRA2B, and RPS24 of SYNCRIP are associated with poor survival (Sun and Yang, 2020). Hence studying alternative splice forms and splicing factors aberrations can be beneficial as targets for therapeutic interventions (Liu et al., 2021).

Known to regulate gene expression by binding to the 3′UTR of the genes, (Cannell et al., 2008), miRNAs contribute to EMT in various types of cancer, including breast cancer, pancreatic cancer, colorectal cancer, prostate cancer, and ovarian cancer. (Burk et al., 2008) (Liu et al., 2013) (Sulaiman et al., 2016) (Wang et al., 2014). The role of upregulated miR-200 has been observed in high-grade and advanced stages of ovarian cancer (Cao et al., 2014; Choi and Ng, 2017) (Choi and Ng, 2017).

In India, the estimated incidence of ovarian cancer is the second highest, next to China, among the world population. India accounts for 76.5% of incidence and 77.5% of mortality of Ovarian Cancer patients among the south central Asian countries (globocan 2020, gco/iarc. fr). The present study aimed to analyse 10 tumour samples in total, 2 normal ovaries, 3 HGSOCs (High-grade serous adenocarcinoma), and one of each ECys (Endometrial Cyst), GCT (Granulosa Cell Tumour), MBOC (Mucinous borderline ovarian tumour), LGSPC (Low grade serous papillary carcinoma) and HGSPC (High grade serous papillary carcinoma), at whole-exome, transcriptome, and miRNA level. (Table 1). We compared genetic alterations in genes and pathways, copy number variations (CNVs), and mutational signatures in patients stratified by histotype, alternative splicing events in different histotypes providing a multi-omic and integrated approach. The population-specific mutations and expression profiles can guide therapy in Indian OVCa.

**TABLE 1:** Patient information.

Sample label	Sample ID	Age	Menarche	Histological subtype	CA125 (U/ML)	Stage	Grade
AA	1105/14	47	14	High grade serous adenocarcinoma (HGSOC)	2074	IV	III
BB	3891/16	-	-	Normal	-	-	-
CC	1157/14	34	14	Endometrial Cyst (ECys)	-	-	-
DD	3842/14	45	14	High grade serous adenocarcinoma (HGSOC)	58.6	II	III
EE	0083/14	35	12	High grade serous adenocarcinoma (HGSOC)	2010	IV	III
FF	5347/14	39	14	Serous Cystadenoma (SC)	212.2	-	-
GG	1121/16	-	-	Normal	-	-	-
HH	1288/14	29	12	Low grade serous papillary carcinoma (LGSPC)	870.8	III	I
II	4211/14	54	12	High grade serous papillary carcinoma (HGSPC)	1582.6	IV	III
JJ	4822/14	52	12	Mucinous borderline ovarian tumour (MBOC)	171.6	-	-
KK	5396/14	57	15	Granulosa cell tumour (GCT)	372.3	-	-
LL	8120/14	57	16	High grade serous adenocarcinoma (HGSOC)	949.4	I	III

## 2 Materials and methods

### 2.1 Study cohort

Here, we analysed the exome and transcriptome data obtained from 10 tumour and 2 normal samples of ovarian cancer patients of Indian origin to understand the underlying molecular mechanism involved. The Ovarian cancer patient samples used for the study were procured from KMIO, Bengaluru, Karnataka, India. The study was approved by the institutional review board (KMIO ethics/006/Dec2015) and written informed consent was obtained from all patients. The tumour tissue and normal samples were collected in RNA later (Ambion, United States).

### 2.2 RNA extraction and library preparation

Total RNA was extracted using RNAiso Plus (Takara, Japan) from tumours and normal samples. Briefly, the tissue was homogenised in RNAiso Plus and 1/10th volume of 2 M sodium acetate and chloroform was mixed vigorously and kept on ice for 10 min and the aqueous phase was collected after centrifugation at 12,000 rpm. RNA was precipitated by adding an equal volume of isopropanol and pelted at 14,000 rpm. The pellet was washed with 80% ethanol and resuspended in DEPC (Sigma-Aldrich, United States) water. RNA was quantitated using QUBIT, and the quality was checked using TapeStation.

mRNA libraries were prepared using RNA Library Prep Kit v2 (New England Biolabs, United States). In brief, mRNA was isolated using oligo-dT beads, followed by fragmentation. Fragmented RNA was then converted to cDNA, and adaptor ligation was performed. Size selection was performed on adaptor-ligated libraries using AMPure beads (Beckman-Coulter, United States). The libraries were amplified and checked on a tape station to determine the library size. The samples were sequenced in-house using Illumina HiSeq 2,500 to acquire 100 bp paired-end reads. Samples had reads >10 million.

### 2.3 Genomic DNA extraction

After RNA extraction, DNA extraction was done using a Back Extraction Buffer (BEB) (Extraction of DNA from TRIZOL preparations). Briefly, BEB was added to RNAiso Plus/TRIzol tubes containing only the interphase and organic (lower) phase of samples after RNA extraction. DNA was precipitated using isopropanol and washed with 70% ethanol. DNA samples were then dissolved in the TE buffer.

### 2.4 Whole exome library preparation and sequencing

To prepare libraries for Whole Exome Sequencing, Agilent SureSelect V6 +UTR was used. 100 ng–1 µg of genomic DNA was sheared with the Covaris S220 (Covaris, Woburn, MA, United States) followed by end-repair, 3′end Adenylation, and ligation with paired-end adaptors. After ligation, enrichment of the DNA library was performed (Desai et al., 2021). Final libraries were checked for quality (fragment size approx. 300–400 bp) and quantity using Agilent Tapestation 2,200 system.

### 2.5 Whole exome sequence analysis

The quality of sequenced reads was checked using FastQC (Wingett and Andrews, 2018); exome reads were trimmed using TrimGalore and aligned to human genome hg38 using Bowtie2 (Langmead and Salzberg, 2012), and variants were called using BCFtools utilities (Li, 2011). Variants observed in normal ovarian tissue and 1,000 genomes (The 1000 Genomes Project Consortium et al., 2015) were subtracted from tumour samples to obtain only tumour sample-specific variants. These variants were further filtered for depth and annotated HIGH and MODERATE impact variants using SnpEff (Reumers et al., 2005) and VEP (McLaren et al., 2016) were considered. For Copy number alterations, CNVkit (Talevich et al., 2016) was used to obtain copy number alterations subjected to filtration of copy number greater or equal to 3 with *p*-value <0.05. Graphs were plotted using heatmap and scatter sub-utilities from the CNVkit tool.

### 2.6 Differential gene expression analysis

Transcriptome reads were checked for quality with FASTQc, adaptors trimmed using TrimGalore, and aligned to GRCh38 using STAR aligner v2.7.10a (Dobin et al., 2013), a splice-aware aligner. For differential gene expression, read counts were obtained using the quant option from STAR and normalised using the Voom package in R (Law et al., 2014) (Supplementary Figure S1). DEGs were obtained using DESeq2 (Love et al., 2014) on tumour samples against normal genes with absolute log2 fold change value > 1 (for upregulated genes) and <−1 (for downregulated genes) and adjusted Benjamini Hochberg *p*-value <0.1 were considered for further analysis.

### 2.7 Identifying gene hubs and interactions using WGCNA and immune profile using CIBERSORTx

WGCNA (Langfelder and Horvath, 2008) was used to construct coexpressed gene modules correlating with the phenotype, and the subtypes of ovarian cancer with the obtained normalised counts. CIBERSORTx (Newman et al., 2019) was used to obtain an immune profile of the tumour and normal samples by comparing them against 22 immune cell fraction types. Modules with Eigen membership correlation with Pearson correlation greater than 0.5 and *p*-value <0.05 belonging specifically to the particular subtype were considered.

### 2.8 miRNA library preparation

Ovarian tumour and normal tissue patient samples were crushed and homogenised in liquid nitrogen. Using RNAiso Plus (Takara, Japan), RNA was extracted from the samples, followed by RNA quality checks; the miRNA library was prepared using miRNA Library Prep Kit v2 and sequenced inhouse to obtain 50 bp paired-end reads using Illumina Hiseq 2,500. Three HGSOC samples (A, E, and L) were used for miRNA library preparation (Table 4), and obtained reads were processed downstream for miRNA seq analysis.

### 2.9 Differential miRNA analysis

For miRNA, after FastQC quality checks, trimming for adaptors using TrimGalore was performed, and reads were aligned using bowtie2 (Langmead and Salzberg, 2012). The differentially expressed miRNAs were determined using DESeq2 and gene feature annotation from miRBase (Kozomara, Birgaoanu, and Griffiths-Jones, 2018). The gene targets were found using TargetScan (Agarwal et al., 2015), and the implications such as differential expression of miRNA targets in TCGA ovarian cancer datasets and the Kaplan Meier survival plots for these target genes were obtained from the GEPIA2 database (Tang et al., 2019).

### 2.10 Alternative splicing analysis

rMATS (Shen et al., 2014) and SUPPA2 (Trincado et al., 2018) were used to find splicing patterns across subtypes. Alternative splice events with absolute psi value >20% and FDR <0.05 were considered for significance. The Uniprot database (The UniProt Consortium, 2018; Trincado et al., 2018) was used to know the effect of protein domains of the spliced forms. Splice graphs were plotted using the ggsashimi package (Garrido-Martín et al., 2018) and GENCODE v41 gene features.

### 2.11 Pathway analysis

PPI interactions were obtained using STRING v11 (Szklarczyk et al., 2018), visualised using Cytoscape (Shannon et al., 2003), and pathway analysis was done using GSEA (Subramanian et al., 2005) and Reactome (Fabregat et al., 2017). The COSMIC database (Tate et al., 2018) was used to obtain the CGC (Cancer Gene Census) gene list.

### 2.12 TCGA and GEPIA analysis

The cBioportal (http://cbioportal.org) was used to explore and analyse multidimensional ovarian cancer genomic data from TCGA (Gao et al., 2013). The frequency of variants and their association with survival was visualised, and compared with its expression and the survival data using the GEPIA2 database, (http://gepia.cancer-pku.cn/), a web based tool. The normal data for expression was obtained from GTEx (Tang et al., 2019).

### 2.13 Statistical test

Statistically significant *p*-val < 0.05 and absolute copy number≥3 were considered for fold copy number analysis in CNVkit. In DESeq2 Benjamini–Hochberg *p*-adj < 0.05 and absolute Log2foldChange >1 was considered. For WGCNA, we used module trait relationship correlation greater than 0.5 and *p*-val < 0.05. For GSEA pathways with FDR < 0.05 and Benjamini–Hochberg *p* adj < 0.05 and NES >0 were considered. In rMATS, a likelihood-ratio test was used to obtain the *p*-value that the difference in the mean exon junction count values between two sample groups exceeds a given threshold. Events with FDR< 0.05 and absolute dpsi (percent splice inclusion change difference) > 0.2 were analysed.

## 3 Results

### 3.1 Clinical characteristics of ovarian cancer patients

A total of 10 ovarian samples in the age range of 29–57 years representing 4 ovarian cancer histotypes were sequenced. One sample each for the histotypes; low-grade serous papillary carcinoma (sample H), high-grade serous papillary adenocarcinoma (sample I), mucinous borderline ovarian carcinoma (sample J), serous cystadenoma (sample F), granulosa cell tumour (sample K) and endometrial cyst (sample C) with CA-125 abnormally high and 4 samples (A, D, E, and L) were HGSOCs (35–57 years). HGSOC samples were grade III, with stages I to IV represented in individual samples (Table 1). The study used two normal ovary samples B and G (ovaries were removed for the presence of benign cysts).

### 3.2 Characterization of exome and CNV in histotypes of Indian ovarian cancer

**FIGURE 1:**
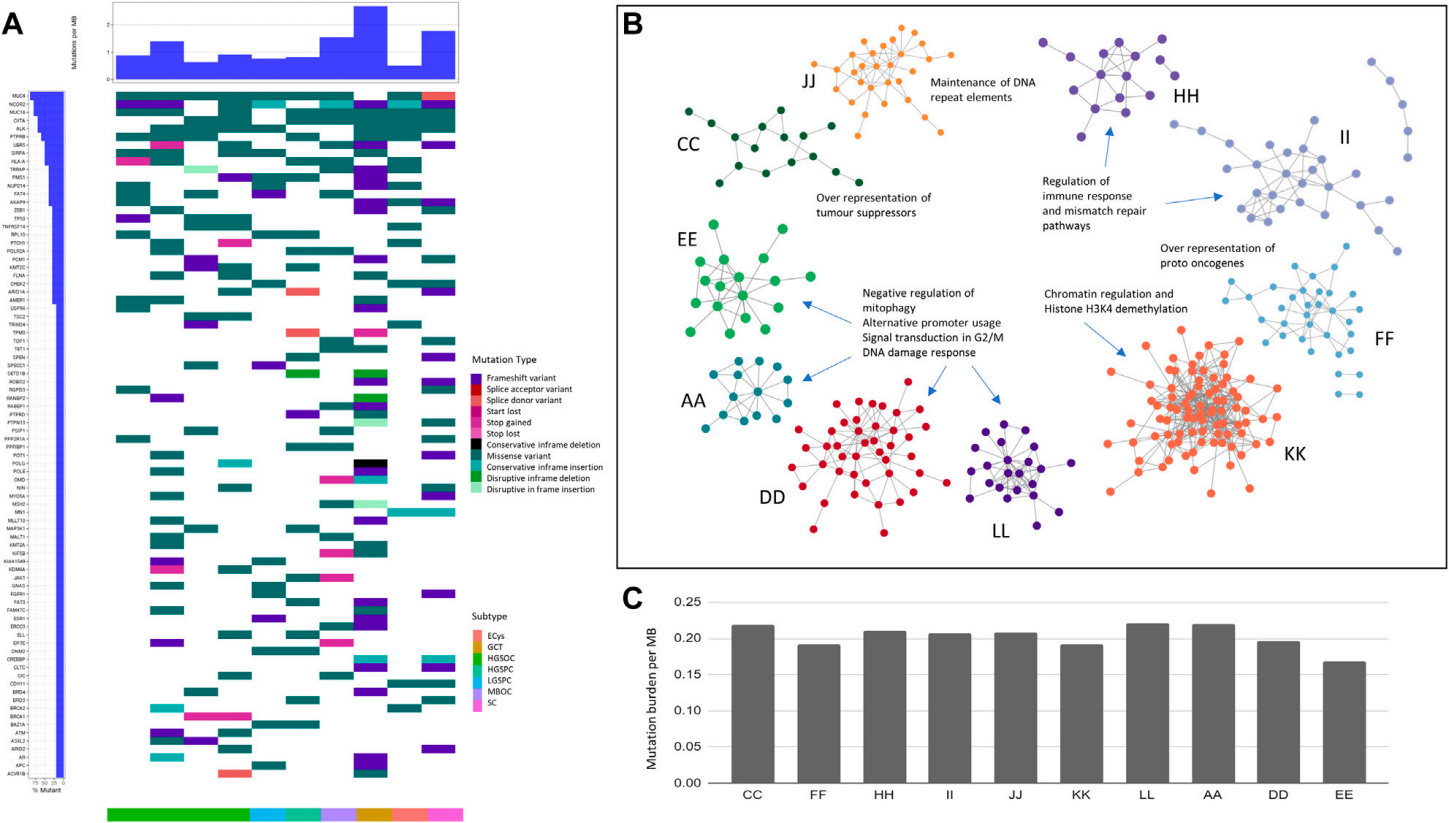
Exome analysis of ovarian cancer histotypes in the Indian cohort. **(A)**. Waterfall plot showing mutations in COSMIC driver genes across ovarian cancer subtypes. **(B)**. Pathways affected by mutations across subtypes. **(C)**. Ovarian cancer samples tumour mutation burden per MB in subtypes.

Whole exome sequencing was performed to compare and contrast the mutations driving oncogenesis in ovarian cancer histotypes. The alignment ranged from 85% to 99% with coverage of 96 to 135X (Table 2). Thousand genome and normal sample variants were subtracted to obtain tumour-specific variants. The tumour mutation burden (TMB) was calculated to check if different histotypes of ovarian cancer have differences in the number of mutations that drive oncogenesis (TMB). A very narrow range was observed across all the histotypes. HGSOCs showed maximum tumour burden (Figure 1C) ranging from 0.22 to 0.175 mutations/Mb, which correlated with age of onset of 45–57 years (samples A and L) vs. 35 years (sample E).

**TABLE 2:** Exome sample details.

Sample	Histological subtype	Alignment percentage (%)	Number of reads	Coverage	Mutation Burden
CC	Endometrial cyst	99.31	54054738	126.6907922	0.2184412894
FF	Serous Cystadenoma	91.73	40978105	96.04243359	0.1917579481
HH	Low grade serous papillary carcinoma	93.73	42860141	100.4534555	0.2103541377
II	High grade serous papillary carcinoma	98.76	41009263	96.11546016	0.206728449
JJ	Mucinous borderline ovarian tumour	97.51	44912839	105.2644664	0.207951465
KK	Granulosa cell tumour	85.43	40034358	93.83052656	0.191501643
LL	HGSOC	95.67	46441479	108.8472164	0.2210474534
AA	HGSOC	99.58	52827386	123.8141859	0.2201298647
DD	HGSOC	98.68	45736444	107.1947906	0.1967586286
EE	HGSOC	93.58	44306471	103.8432914	0.1688746549
BB	Normal	94.66	49248427	115.4260008	0.1835175771
GG	Normal	96.62	57882700	135.6625781	0.1908374465

To identify tumour-specific variants, bcftools utilities were used, and 253,158 mutations from the ovarian tumour samples were obtained. A total of 150,448 were coding variants across tumours, of which 45% were missense, 24% synonymous, 16% frameshift, 7% inframe insertions, 5% stop gains, and 1% inframe deletions. Of 45% of missense variants, 3.7% were damaging variants. Among the variants obtained, 6,764 variants have been reported in the COSMIC database. 1,588 high-impact variants in genes classified as probably damaging and deleterious to variants of unknown significance (VUS) were observed, from which some are population specific detailed in (Supplementary Table S1).

Granulosa cell tumour sample (GCT) (sample K) in the Indian cohort harboured high-impact frameshift variants in the transcription factor family of FOX genes, namely, FOXG1 and FOXC2. FOXL2 c.402C>G somatic mutations were absent in the Indian cohort, observed in ∼95% caucasian granulosa tumours (Shah et al., 2009) (Nolan et al., 2017) (Roze et al., 2020). High TMB in HGSOC (sample D) and GCT (sample K) harboured the POLE mutation in the Indian cohort, similar to previous reports (Wang et al., 2018).

Further analysis of the variants in cancer census genes, oncogenes, and tumour suppressors across histotypes led to identifying common and unique variants. The most mutated COSMIC genes across all samples were MUC4 (9/10), NCOR2 (8/10), MUC16 (8/10), and CIITA (8/10) (Figure 1A). Novel mutations in these genes were recorded. Other than the ones mentioned above, most mutated oncogenes across samples were ALK (7/10), UBR5 (5/10), and tumour suppressors, PTPRB (6/10), and SIRPA (5/10), respectively. (Supplementary Table S1).

Ovarian cancer is known to harbour aberrations in DNA damage response genes (Tomasova et al., 2020), especially homologous recombination genes, resulting in HRD (Homologous recombination deficiency). We observed mutations in BRCA1, BRCA2, RAD51D, PRKDC (2/10 samples), TP53, APLF, CHEK2 (3/10), and PMS1 was the most mutated (4/10). Likely pathogenic mutations in RAD51B, a member of the RAD51-XRCC2 (BCDX2) complex in HR, were observed in the serous cystadenoma sample. The high-impact novel population-specific mutations in TP53: The guardian of the genome, were observed at high frequency (6/10) samples. Other high-impact mutations were observed in POLQ (3/10) and ATM (4/10) (Supplementary Table S1). Apart from the genes involved in HR, mutations in mismatch repair, nucleotide excision repair, and Base excision repair (BER) were observed. These mutations in DNA damage repair and response genes may further cause genomic instability.

Cataloguing of moderate and high impact mutations in individual samples showed the most number of variants (106) in granulosa cell tumour (sample K), 49 variants in serous cystadenomas (sample F), and 48 in HGSOC (D). In contrast, HGSOC A, E, and L samples showed 21, 23, and 31 variants, respectively. Granulosa tumours are rare (Roze et al., 2020) and harboured low-impact mutations in AR and ESR1, the genes for steroid hormone receptors in the Indian Cohort, and had the largest number of variants (Figure 1A), contrastingly MBOC and GCT did not show any pathogenic variants.

The histotype-specific mutational signatures were recorded, and we classified SNVs of moderate and high impact based on SNPEff and VEP annotations, and further into oncogenes (ONC) and tumour suppressors (TSGs). The highest number of TSGs (21) and oncogenes (12) were altered in the granulosa cell tumour (sample K), followed by serous cystadenoma (sample F) with TSG and oncogenes (14 and 9), respectively. In HGSOCs, mutations in TSG (8–16) and oncogenes (2–9) were observed across samples. The least number of TSGs (8) and oncogene (4) was observed in the endometrioid cyst (sample C). From the analysis of variants, heterogeneity was highlighted within and between ovarian cancer histotypes (Supplementary Table S1).

Unlike the TCGA (Network and The Cancer Genome Atlas Research Network, 2011) cohort, where 96% of the ovarian cancer samples showed TP53 mutation, TP53 mutation was observed only in 50% of HGSOC (2/4) samples in the Indian Cohort.

To check whether histotype-specific variants in the oncogenes and TSGs correlated with unique altered pathways, which can provide mechanistic insights, pathway analysis using PPI (protein-protein interactions) was performed using the STRING v11 database (Figure 1B).

In HGSOCs, negative regulation of mitophagy, alternative promoter usage, and signal transduction involved in G2/M DNA damage response was enriched. In granulosa cell tumours, the altered pathway was chromatin regulation and Histone H3K4 demethylation. In high and low-grade papillary adenocarcinoma, the regulation of immune response and mismatch repair pathways were altered (Figure 1B). Maintenance of DNA repeat elements and chromosomes was the significantly altered pathway in mucinous borderline cancer (Figure 1B).

**FIGURE 2:**
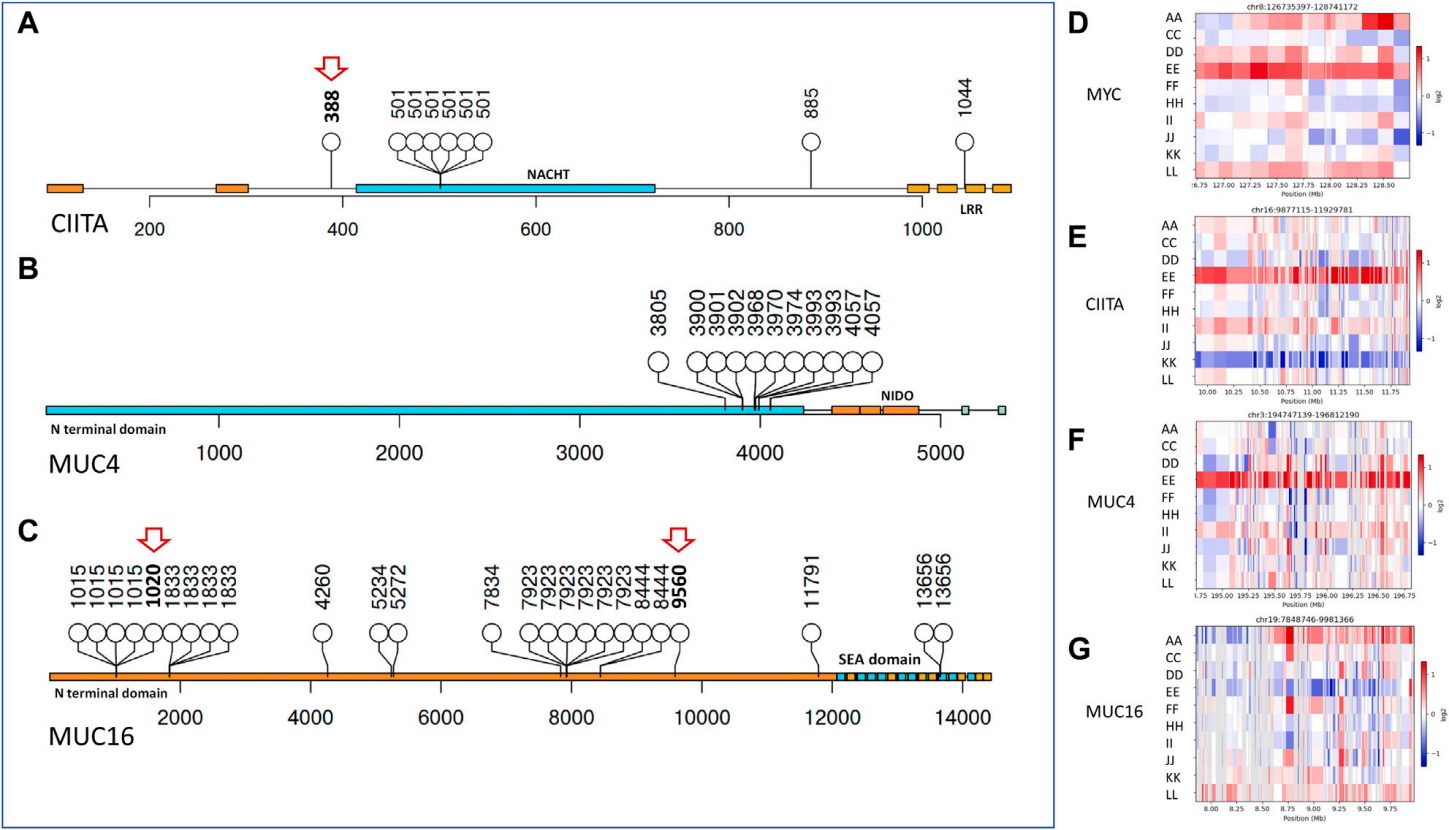
Copy number variants and affected protein domains of impacted genes. **(A–C)** Lollipop plot showing mutations affecting protein domains (in blocks) and their frequency in CIITA, MUC4, and MUC16, respectively, novel mutations indicated in red arrows. **(D–G)** Copy number alteration in MYC, CIITA, MUC4, and MUC16 across ovarian cancer subtypes (Red showing copy number gain and blue signifying copy number loss). Sample E showing most copy number gains in the given genes.

Copy number variant (CNV) analysis (Figures 2D–G) revealed significant events to be copy number gains, with most in HGSOC (265, 206, 96, 77) in samples (I, E, L, A), respectively. No significant CNV was observed in endometrioid cyst (sample C), serous cystadenoma (sample F), and mucinous borderline (sample J) samples. Although granulosa cell tumours showed most SNVs, CNVs were limited. A similar observation was made by Roze et al. in the largest cohort of GCT studied to date (Roze et al., 2020). Interestingly, no mutation was observed in BRCA1 and BRCA2, coinciding with a lack of structural variants.

#### 3.2.1 Mutation and copy number variations in CIITA, MUC16, and MUC4 drive oncogenesis in histotypes of Indian ovarian cancer

In most HGSOCs reported, TP53 is the most mutated, which was not observed in the Indian Ovarian Cancer cohort. On the other hand, homologous recombination deficiency (HRD) in HGSOCs was observed in the Indian and TCGA cohorts (The Cancer Genome Atlas Program, 2018). The Indian Cohort showed MUC16, MUC4, and CIITA to be most mutated and carry CNV in the same genes apart from MYC (Figures 2D–G), which is known to be amplified in most cancers, including ovarian cancer (Zhou et al., 1988). To understand if these genes could be drivers of oncogenesis in ovarian cancer histotypes, we checked if the genes carried hotspots for mutation or were dispersed across the entire gene. MUC4 mutations clustered near its NIDO domain, CIITA mutations in the LRR domain, and the NACHT domain, whereas MUC16 mutations were observed in clusters distributed across its length. Population-specific novel mutations in CIITA at c.1162T>A (p.Trp388Arg) and in MUC16 c.3059_3060insGATAGA (p.Thr1020_Ile1021insIleAsp) and c.28678_28679insTTA (p.Val9560_Ser9561insIle) were found. Mucins are cell surface receptors with cytoplasmic adaptor proteins to help them participate in signal transduction. With cancer cells known to express aberrant forms and amounts of mucins, mutations in these might provide tumorigenic advantage and help them proliferate. (Hollingsworth and Swanson, 2004). Whereas, CIITA being an MHC II transactivator, mutations might cause hindrance in MHC II expression and cancer immunity (Son et al., 2020).

Figure 2 shows the mutation distribution in all three genes. Notably, in CIITA, the same mutation was observed in 6/10 (60%) samples in the NACHT protein domain. The consequences of a single mutation in the NACHT domain indicate the selection for survival of the cells and as a potential driver (Kandoth et al., 2013). In contrast, 7.81% of the samples show a mutation in the CIITA gene in the TCGA cohort, the high incidence in the Indian cohort can be attributed to low sample numbers but the presence is not restricted to HGSOC, which is the most studied in other populations, making CIITA as driver irrespective of the histotype. Further CNV analysis indicated CIITA copy number gain in 2/10 Indian cohort samples. In contrast, NCOR2, a nuclear receptor corepressor involved in transcriptional silencing, was mutated in 8/10 (80%) samples irrespective of the histotype in the Indian Cohort and 14.06% in the TCGA cohort.

About 73.44% of the samples showed mutations in MUC16 and 15.63% in MUC4 in the TCGA cohort. Contrastingly, 90% of the samples showed a mutation in MUC16 and 80% in MUC4 in the Indian Cohort. Signs of selection were also evident in the MUC16 genes, where 6/10 individuals showed mutation at the same position in the gene and consequent amino acid. Several mutation clusters of selection were observed in the MUC16 gene. A similar analysis with MUC4 showed a very distinct cluster in the N terminal region close to the NIDO domain, which is known to play a role in tumorigenesis (Xia et al., 2017).

Interestingly, one of the HGSOC samples, sample E showed amplification in MUC4, CIITA, and MYC but not MUC16 (Figures 2D–G), knowing that gene amplification might indicate an increase in the amount of RNA or protein of the gene, it would be interesting to know its transcriptome profile. In contrast to the above, the granulosa cell tumour (sample K) showed no amplification in MYC and CIITA, with subtle amplification in MUC16 and MUC4. We also checked for MYC amplification in the samples and found MYC to be amplified in high grade samples. From the above, MUC4 is known to be an active player in breast cancer metastasis and its deregulation has been implicated in gastric adenocarcinomas through the activation of ERBB2/HER2 and ERBB3/HER3 receptor kinases (Dreyer et al., 2021) (Funes et al., 2006) (Senapati et al., 2008).

Hence, the frequency and hotspot mutations in the genes NCOR2, CIITA, MUC4, and MUC16 point towards their role in driving oncogenesis in all ovarian cancer histotypes. Patient and histotype-specific mutation signatures were observed in the Indian Cohort (Figures 2A–C), which requires validation in a large cohort. The mutation analysis across the histotypes and patients revealed heterogeneity of the tumour samples.

### 3.3 WGCNA of the Indian ovarian cancer revealed pathways unique to histotypes

**FIGURE 3:**
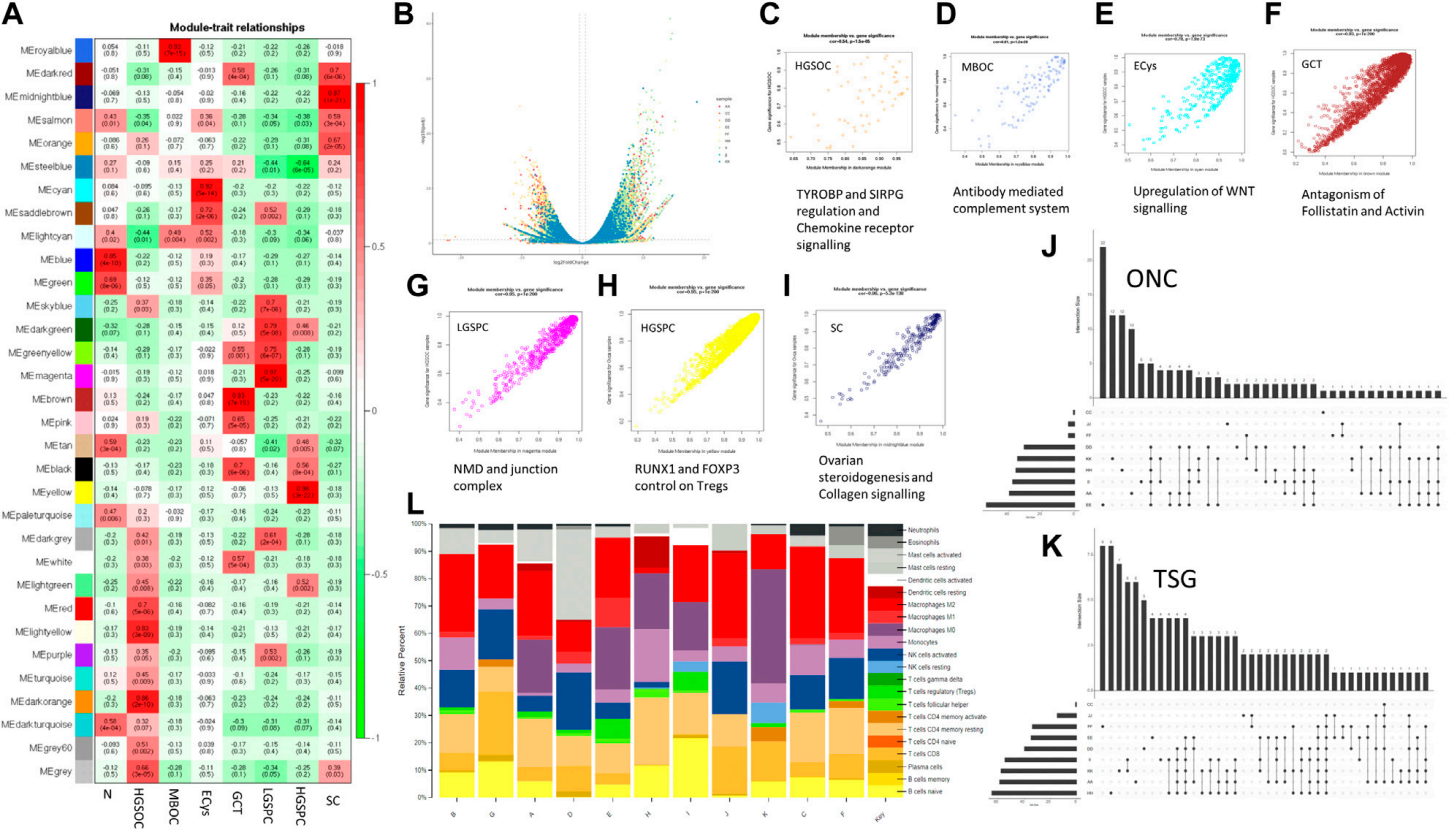
Transcriptome analysis of ovarian cancer histotypes. **(A)**. WGCNA module trait relationship between modules and subtypes (red showing positive correlation with the trait, i.e., the subtype and green showing negative correlation for the same) **(B)**. Volcano plot showing expression distribution along with significance for the samples. **(C–I)** Scatter plot of module trait relationship corelation and its affected pathways for HGSOC, MBOC, ECys, GCT, LGPSC, HGPSC, and SC, respectively. **(J)**. Upregulated oncogenes (ONC) across subtype samples. (Each dot indicates the intersecting set and *y*-axis bar indicating intersection size) **(K)**. Downregulated tumour suppressors (TSG) across subtype samples. (Each dot indicates the intersecting set and *y*-axis bar indicating intersection size) **(L)**. CIBERSORTx immune profile of OVCa samples across 22 immune cell subtypes.

A whole transcriptome analysis evaluated the patient and histotype-specific gene expression signatures. 96%–98% alignment was observed across samples (Tumour and Normal) except in low-grade papillary carcinoma, which was 63% with coverage of ∼×100 (Table 3). A volcano plot depicting differentially expressed genes across samples showed roughly equal numbers of significantly up and downregulated genes in ovarian cancer histotypes (Figure 3B). A MAplot has been plotted for the same (Supplementary Figure S3). Further, a cutoff of LFC (log2 fold) >5 and padj<0.01 highlighted sample-specific changes in gene expression to get a view of alterations in individual samples. (Table 4). In HGSOCs, more genes were upregulated than down. However, each of the samples varied in the number of genes indicative of sample-specific changes, which did not have much correlation with the grade, but with stage, where stage IV showed a more considerable change in significantly regulated genes vs. stage I (∼1,200 vs. 595 DE genes), respectively. High-grade serous papillary and borderline mucinous carcinoma showed the opposite trend, where more genes were downregulated than upregulated (Table 4). The least Differentially expressed (DE) genes were observed in endometrioid cysts, followed by mucinous borderline carcinoma (sample J).

**TABLE 3:** Transcriptome sample details.

Sample	Histological subtype	Alignment percentage (%)	Number of reads	Coverage
CC	Endometrial cyst	96.55	83482473	195.6620461
FF	Serous Cystadenoma	98.38	96633956	226.4858344
HH	Low grade serous papillary carcinoma	63.14	106366467	249.296407
II	High grade serous papillary carcinoma	96.37	12076661	28.30467422
JJ	Mucinous borderline ovarian tumour	98.02	111522756	261.3814594
KK	Granulosa cell tumour	98.53	74894026	175.5328734
AA	HGSOC	97.84	72431084	169.7603531
DD	HGSOC	97.84	84552927	198.1709227
EE	HGSOC	98.73	91884592	215.3545125
BB	Normal	98.82	102375941	239.9436117
GG	Normal	98.49	169076029	396.271943

**TABLE 4:** Differentially expressed genes amongst the OVCa samples.

Sample	Upregulated genes (LFC > 5, padj < 0.01)	Downregulated genes (LFC > 5, padj < 0.01)
AA	778	477
CC	3	3
DD	490	105
EE	660	564
FF	101	17
HH	667	219
II	242	316
JJ	11	27
KK	402	249

To understand more about DE genes in the tumourigenic context, we used literature and COSMIC-curated TSGs and Oncogenes. We identified the most downregulated TSGs with significant changes in gene expression (Figure 3K). We intersected upregulated oncogenes and downregulated TSGs, respectively across samples (Figures 3J, K) to find commonly dysregulated oncogenes and TSGs. Larger intersections were observed in downregulated TSGs, indicating similar downregulated gene strategies and distinct oncogene activation amongst tumours and subtype samples. Low-grade papillary carcinoma (sample H) showed most TSGs downregulated and the least in endometrioid cysts (sample C). Similarly, upregulated oncogenes were higher in HGSOCs than low-grade papillary and granulosa cell tumours (Figure 3J). The common downregulated TSGs in all samples except endometrioid cysts are PER1 and ZNF331 (9/10). Period circadian protein homolog 1 (PER1) is an important component of the circadian clock and regulates carcinogenesis. OVCa patients with low PER1 expression had a reduced overall survival rate and poor prognosis (Chen et al., 2021). PER1 is also known to modulate anticancer drug response (Bellet et al., 2021). PTPRD (7/9 samples) and CD11 (6/9 samples) were downregulated (Supplementary Table S1). CD11 is known to regulate cell adhesion, downregulation might lead to enhanced migration (Wang et al., 2015). Among common oncogenes, ERBB3, ERBB4, ETV4, and SYK were upregulated in 6/9 samples (Supplementary Table S1). Breast cancer cells that make too much HER2/ERBB2 spread faster but are also more likely to respond to HER-specific chemo and immunotherapy, with mutations in MUC4 known to associate with upregulated ERBB proteins, observed in the Indian cohort (Mill et al., 2011). ERBB3 and ERBB4 have been shown to be upregulated in chemoresistant ovarian cancer and upregulation was observed in Indian ovarian cancer, which might serve as a potential therapeutic target.

We performed weighted gene co-expression network analysis (WGCNA) and identified significant modules, indicating sample and histotype-specific gene co-expression. The modules were selected with *p*-value significance (*p* < 0.05) and their highest module-trait relationship unique to the histotype sample, ranging from 0.7 to 0.98, and gene significance module membership with R > 0.55 (Figure 3A). To Identify interacting genes in coexpressing modules, pathway analysis was performed using Reactome (Figures 3C–I) (Table 5).

**TABLE 5:** WGCNA curated module pathways for the sample histotypes.

Sample histotypes	Pathways
Serous Cystadenoma	Ovarian steroidogenesis and Collagen receptor signalling
Endometrial cyst	Upregulation of Wnt signalling
Granulosa cell tumour	Antagonism of follistatin and activin
HGSOC	TYROBP and SIRPG signalling, Regulation and Chemokine receptor signalling (CXCL3 and CXCL1)
Mucinous borderline ovarian tumour	Antibody mediated complement system
Low Grade Papillary Serous Papillary carcinoma	NMD exon Junction complex
High Grade Serous Papillary carcinoma	RUNX1 and FOXP3 control in development of Tregs

Collagen receptor signaling and steroidogenesis were enriched in Serous Cystadenoma. Differences amongst histotypes in the distribution of collagen I was also observed in cystadenomas, borderline tumours, and different grades of OVCa (Davidson et al., 2014) (Zhu et al., 1995). Known to promote the maintenance of cancer stem cells *via* EMT, upregulation of Wnt signaling was observed in Endometrial cyst samples. Whereas, Granulosa cell tumours showed enrichment of antagonism of Activin and Follistatin in the Indian Cohort. Follistatin antagonises activin, which is overexpressed in OVCa, with its levels in turn regulated by BRCA1 (Karve et al., 2012). Activin inhibitor STM 434 is in Phase I clinical trial for granulosa cell tumour (Tao et al., 2019).

In HGSOCs, immune-related TYROBP and SIRPG signaling and Chemokine receptor signaling pathways were enriched. In TME (Tumour Micro Environment), the role of TYROBP and SIRPG in regulating NK cells, leukocyte adhesion, and modulating immune cells is well established. (Yu et al., 2021). Mucinous Borderline ovarian cancer showed enrichment in the complement system pathway. Complement-associated proteins can act as antagonists and increase tumour cell proliferation, migration, and invasion and induce angiogenesis, as observed with antagonists of C5aR1 and C3aR (Cho et al., 2014). In such cases, anti-VEGF therapy has shown tumour reduction in patients and animals. EJC-mediated NMD was the pathway specific to the LGSPC sample. The EJC (Exon Junction Complex) mediated NMD (Non-sense-mediated decay) is involved in alternative Splicing, mRNA processing, and decay. Tumours exploit the mRNA processing with aberrant expression and mutations in key regulators, this helps them adapt to their microenvironment, to cause the destruction of key tumor suppressor mRNAs and promote the transcription of erroneous ones (Popp and Maquat, 2018). Whereas FOXP3 and RUNX1 control of T regs was observed in HGSPC. In OVCa, CD8+ve T cells are indicators of a good prognosis vs. Tregs as a bad prognosis (Curiel et al., 2004). Hence it would be interesting to understand the immune profile of samples to check for implicated immune pathways from above.

#### 3.3.1 Immune profiling of ovarian cancer reveals histotype-specific immune cell enrichment

We observed that in the modules obtained from WGCNA, immune pathways were enriched in HGSOC, MBOC, and HGSPC; we performed CIBERSORTx to classify histotypes based on immune profiling. We profiled 22 immune cell fractions using gene expression data of normal and tumor samples. HGSOCs (sample A, D and E) showed a relatively lower fraction of naive B cells than normal and lower than that observed in high-grade serous papillary carcinoma. Sample and histotype-specific changes in immune cell fraction were observed (Figure 3L). Across histotypes, HGSOC, LGSPC, and GCT showed significantly high M0 macrophage. Tregs were high in HGSOC and HGSPC samples. Resting memory T cells were enriched in LGSPC. In OVCa CD8+ve T cells are indicators of a good prognosis vs. Tregs as a bad prognosis (Curiel et al., 2004), hence knowing about T cell fraction may help prognostically and well as designing therapeutic strategy. NK cells resting were observed in MBOC and GCT. Neutrophils were high in ECys, and eosinophils in SC. Mast cells were high in HGSOC (sample D), and M1 macrophage in HGSOC (sample E). Among the HGSOCs, the immune profile varied across the samples. Interestingly normal ovarian samples showed enrichment of M2 macrophage, which has been documented (Ardighieri et al., 2014; Zhang et al., 2020). Among the samples from transcriptome analysis, PER1 was the most downregulated TSG in the Indian cohort except for serous cystadenoma and from literature, its levels correlate with infiltrating neutrophils, regulatory T cells, and M2 macrophages (Chen et al., 2021), which was evident in the Indian cohort (Figure 3L). Overall, each sample had a unique immune profile indicating the need to administer immunotherapy based on the immune cell expression and infiltration. We also checked for the expression of immune checkpoint markers, namely, PD-1 (PDCD1), PD-L1 (CD247), and CTLA4. PD-1 was upregulated in HGSOC (sample E) and CTLA4 and PD-L1 were downregulated in HGSOC (samples A, D), and SC (sample F). Since the sample number of different histotypes of OVCa was a limiting factor, we performed further analysis on HGSOCs.

#### 3.3.2 Upregulated miR-200 family and miR-1269 mark HGSOCs

**FIGURE 4:**
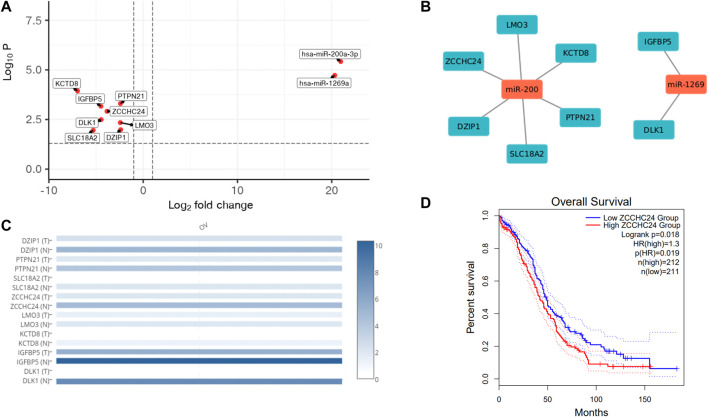
miRNA analysis of HGOSC samples in the cohort. **(A)**. Upregulated miRNAs and their targets in OVCa HGSOC samples. **(B)**. Gene interaction network of miRNAs and their target genes. **(C)**. Expression of miRNA targets in the OVCa TCGA data show similar pattern as observed in our samples. **(D)**. Kaplan Meier plot of ZCCHC24 (miR-200 target), signifying survival probability between high vs. low expressed groups from GEPIA.

We also profiled miRNAs in HGSOC and normal ovarian tissue samples to check for differential miRNA expression contribution to gene regulation leading to carcinogenesis. The miRNA seq of 3 HGSOC (sample A, E and L) and 2 normal samples (sample B and G) was performed, where the alignment ranged from 93% to 98%, and at least 40 million mapped reads (Table 6). We obtained 700 differentially expressed miRNAs, of which only 28 miRNA met the criteria of the adjusted *p*-value of (padj ≤ 0.1) (Table 7). Only 4 upregulated miRNA were obtained when (padj<0.01) and no downregulated miRNA were observed that met the criteria. We extracted genes targeted by miRs using Targetscan and found 6 genes (DZIP1, PTPN21, SLC18A2, ZCCHC24, LMO3, KCTD8) whose expression correlated with upregulation of miR-200a-3p and 2 genes (IGFBP5 and DLK1) which correlated with upregulation of miR-1269 (Figures 4A, B) in the Indian cohort. Expression of the mir200 family has been reported in a study to be high as compared to benign (Savolainen et al., 2020).

**TABLE 6:** miRNA sample details.

Sample	Histological subtype	Number of mapped reads	Alignment percentage
AA	HGSOC	13885291	96.49%
LL	HGSOC	4118074	93.44%
EE	HGSOC	15474531	98.37%
BB	Normal	19701544	96.70%
GG	Normal	15060887	95.99%

**TABLE 7:** Differentially expressed miRNAs in HGSOCs.

Downregulated miRs	Upregulated miRs
hsa-miR-100-5p	hsa-miR-335-3p
hsa-miR-195-3p	hsa-miR-96-5p
hsa-miR-125b-5p	hsa-miR-375-3p
hsa-miR-195-5p	hsa-miR-615-3p
	hsa-miR-582-3p
	hsa-miR-200c-3p
	hsa-miR-7974
	hsa-miR-1269a
	hsa-miR-429
	hsa-miR-200a-3p
	hsa-miR-449b-5p

Additionally, we checked for the expression of these targeted genes in the TCGA cohort using GEPIA (Figure 4C). We were surprised to see a similar pattern in the Indian Cohort. The tumour samples had lower expression of all the genes than normal, indicating tumour suppressor roles of the genes and the oncogenic role of miRs. We further checked if any of the genes could be used to predict survival in the TCGA cohort. ZCCHC24 is a gene known to bind to RNA and regulate RNA splicing (Wang et al., 2021), also correlated with immune cell infiltration in lung and stomach cancer (Huang et al., 2021), whose expression levels significantly correlated with poor survival (Figure 4D).

### 3.4 Integrated mutation and expression analysis of HGSOC revealed upregulation of mutated oncogene and downregulation of mutated TSGs

It is known that a gain of function mutation in proto-oncogene and loss of function in TSG drives cancer. To check whether mutated oncogene and TSGs (drivers of oncogenesis) correlated with expression, we performed an integrated analysis of exome and transcriptome in HGSOCs. Independent mutation or expression studies fail to provide a complete insight into cancer development and resistance, hence we focused on the known mutated TSG and oncogenes in the Indian cohort which were differentially expressed (log2FC > 1.5, padj<0.05).

**FIGURE 5:**
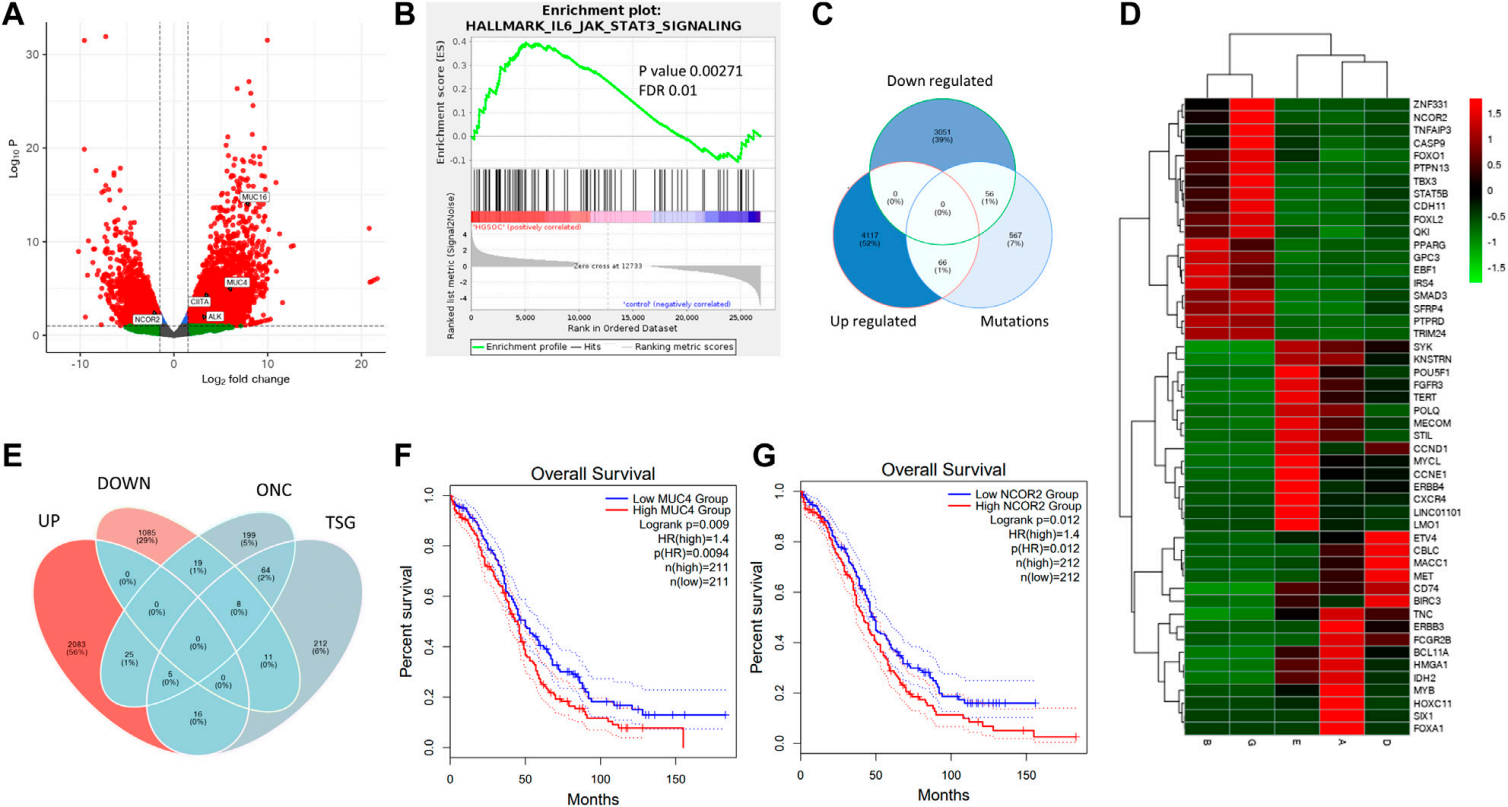
Integrated exome and transcriptome analysis of HGSOCs. **(A)**. Gene expression volcano plot for HGSOCs and highlighted genes represent mutated and significantly differentially expressed ones. **(B)**. JAK-STAT pathway enrichment in HGSOCs with pval 0.00271 and FDR 0.01. **(C)**. Venn diagram showing number of upregulated, downregulated and mutated genes and their respective intersections. **(D)**. Heatmap of common downregulated TSGs and upregulated ONCs. (Red showing upregulation and green indicating downregulation) **(E)**. Venn diagram of differentially expressed genes being ONCs and TSGs in HGSOC. **(F, G)** Kaplan Meier plots of MUC4 and NCOR2 signifying survival probability between high vs. low expressed groups from GEPIA database.

In HGSOC samples (samples A, D, E and L), 7% (567) of the genes were mutated, whereas 39% (3,051) of the genes were downregulated. Only 1% of the mutated genes (56) were downregulated. 52% (4,117) of the genes were upregulated, of which only 1% was mutated (66) (Figure 5C). There were 19 TSGs, and 31 oncogenes downregulated and upregulated, respectively, in HGSOC samples (Figure 5E). It is evident from the volcano plot (Figure 5A) that highly mutated genes MUC4, MUC16, CIITA, and ALK are mutated and overexpressed, of which ALK and CIITA are cancer census genes, whereas NCOR2 a transcriptional corepressor was mutated and downregulated (Figure 5A). CDKN2A, a tumour suppressor, is mutated and upregulated. Similarly, drivers of oncogenesis in breast cancer and AML, IRS4, and MN1 are mutated and downregulated. (Figure 5D) (Ikink and Hilkens, 2017) (Heuser et al., 2007). Since a number of genes were mutated and differentially expressed, we checked whether it correlated with survival using the TCGA cohort. From this, we found that high expression of MUC4 and NCOR2 correlated with low survival (*p* = 0.0094 and *p* = 0.012), respectively (Figures 5F, G).

In the Indian Cohort, NCOR2 was mutated and expressed at lower levels in HGSOCs, indicating population-specific variation. We observed that typical cancer drivers such as BRCA1, BRCA2, and TP53 did not reveal significant differential expression; therefore, we checked for their expression in the non-significant list and BRCA1 and TP53 were upregulated with log2FC of 0.4 and 0.8, respectively with padj<0.05, and BRCA2 although non-significant (padj>0.05) showed log2FC = 2. Whereas non-significant downregulation of BRD4 and ATM was observed in HGSOCs. HGSOCs are deficient in Homologous recombination and mutation of the HR genes (Creeden et al., 2021) (da Cunha Colombo Bonadio et al., 2018), along with expression confirmed the same in the Indian Cohort. Aberrantly expressed genes among HGSOCs showed the indicated transcriptional regulation of pluripotency (POU5F1, MYCL, MYB, TERT) along with upregulated and downregulation of senescence-associated heterochromatin (PEG3, DIRAS3, DLK1), to be impacted. The GSEA analysis of HGSOCs revealed enrichment in the JAK-STAT pathway (Figure 5B), further highlighting its role in immune signaling. On integration, genes returned upregulated pluripotency and downregulated senescence-associated chromatin organization as the hallmark drivers of HGSOCs in the Indian Cohort, as a strategy of tumour cells in supporting their survival. We obtained similar pathways from the mutation analysis. However, most of the genes associated with mutations were not differentially expressed and the DE genes were not much mutated. Therefore understanding both mutation and expression is necessary to identify the control mechanisms of cancer.

### 3.5 Profiling alternative splicing in ovarian cancer histotypes

From pathway analysis of the mutated genes and miRNA-regulated genes, we observed that RNA splicing and metabolism were altered in the Indian cohort. Hence, we performed alternative splicing analysis to identify changes in the spliceosomal genes and aberrant splicing in histotypes of ovarian cancer.

**FIGURE 6:**
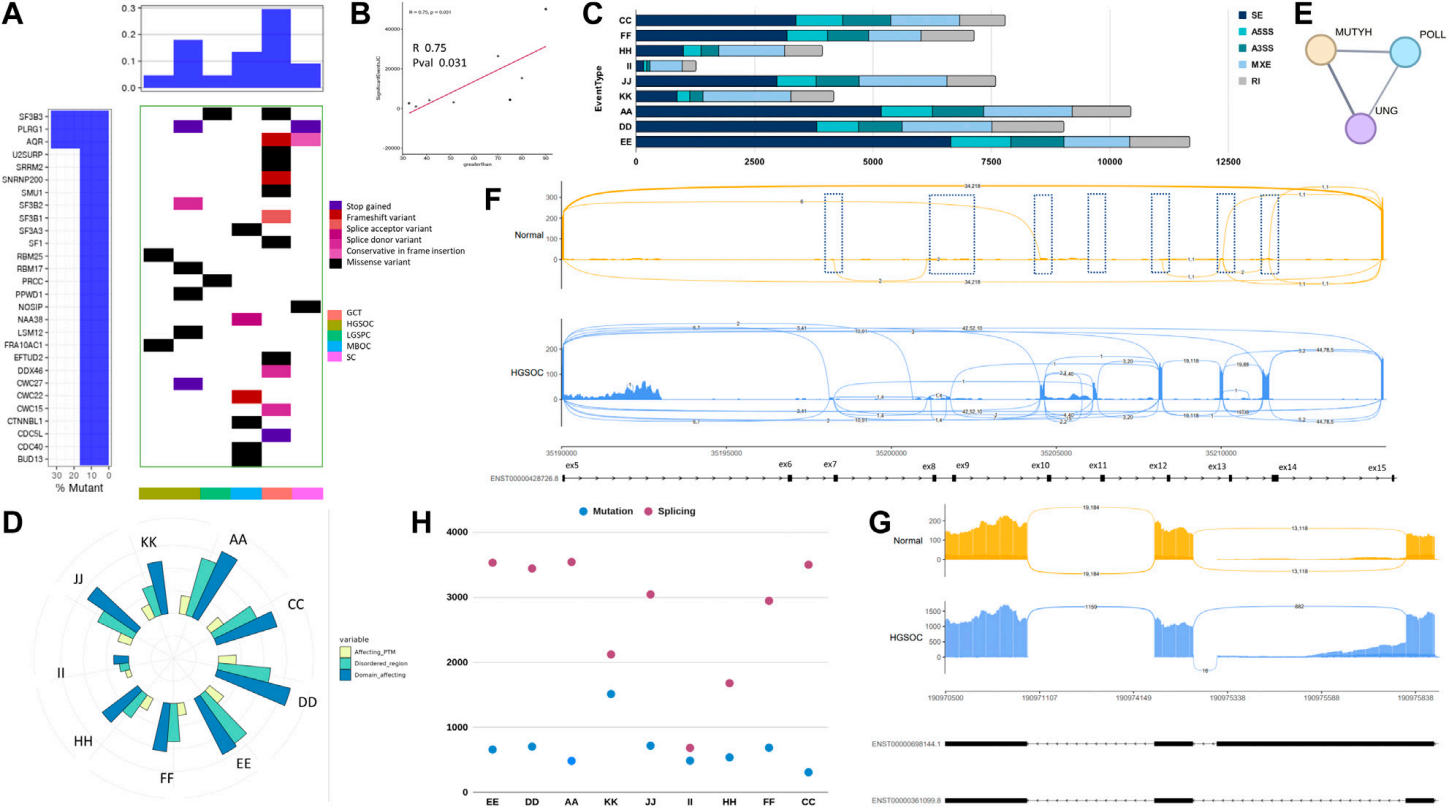
Splicing analysis of OVCa histotypes. **(A)**. Waterfall plot showing mutations in splice factors in patient samples. **(B)**. Corelation plot of upregulated splice factors and number of alternatively spliced events. **(C)**. Significant alternatively spliced events and their abundance in types across samples. **(D)**. Splice events affected protein domains and modifications mapped from Uniprot in OVCa subtypes. **(E)**. Active site spice events affecting Base excision repair pathway. **(F)**. CD44 ex5-ex14 variable region usage in normal and HGSOC samples. Normal samples skip these exons while HGSOCs show variable inclusion events to give rise to oncogenic forms of CD44. Regions indicated in dashed boxes. **(G)**. STAT1 alternative 3′ splice event of a exon in HGSOC harbouring phosphorylated residues involved in signalling. **(H)**. Number of genes altered by mutation and splicing across samples.

All the alternative splice events were analysed to check whether the type of alternative splicing events was histotype specific and whether the splice event was differential across samples. We used rMATS and kallisto for studying the splicing profile across samples. From the analysis, the most abundant alternative splicing event was Exon skipping in HGSOC, HGSPC, ECys, and MBOC (Table 8). In contrast, in LGSPC and GCT histotypes, alternative mixed exon exclusion was the most prevalent type (Figure 6C). HGSOC showed the most splice events individually, with sample A, D and E showing 10,443, 90,223, and 11,677 significant alternatively spliced events. The least splice events were in high-grade papillary carcinoma, with 1,264 events and LGSPCs. From the observed splice events, more than 70% of splice events were as in genes reported by the Spladder project in SpliceSeq (Ryan et al., 2016).

**TABLE 8:** Number of splicing events and mutated splice factors per sample.

Samples	Skipped exon event (SE)	Alternative 5’ splice site event (A5SS)	Alternative 3’ splice site event (A3SS)	Mutually exclusive exons event (MXE)	Intron retention event (RI)	Mutated splice factors
AA	5174	1092	1075	1873	1229	-
CC	3373	996	1008	1458	951	-
DD	3816	882	916	1903	1506	EFTUD2, FRA10AC1, RBM25
EE	6651	1265	1124	1385	1252	CWC27, PLRG1, LSM12, PPWD1, RBM17, SF3B2
FF	3,194	850	873	1,109	1,099	PLRG1, AQR, EFTUD2, NOSIP
HH	1,009	364	372	1,400	783	EFTUD2, PRCC, SF3B3, SPPL2B
II	168	57	73	683	283	EFTUD2, HYPK
JJ	2,976	834	897	1862	1,012	CWC22, NAA38, BUD13, CDC40, CTNNBL1, SF3A3
KK	865	274	280	1856	890	AQR, CDC5L, ORC1, SF3B1, SNRNP200, CWC15, DDX46, EFTUD2, SF1, SF3B3, SMU1, SPPL2B, SRRM2, U2SURP

To understand if the number of splicing events correlated with mutations or the expression of splicing proteins, we plotted the expression of spliceosome proteins with splicing events. We observed a significant correlation of 0.75 and *p*-value = 0.031 (Figures 6A, B) indicating a strong correlation between the upregulation of splice factor genes and observed splice forms. When checked for correlation of splice events solely with splice factor mutations, no correlation was observed. Studies have shown that SF3B1, SF3B2, and SF3B3 can regulate target gene expression by alternative splicing to promote cancer progression (Chen et al., 2017), also SF3B3 controls AS in renal cancer and SF3B4 in ovarian cancer by regulating AS of RAD52 (Diao et al., 2022). Mutations in these spliceosome complexes were observed in the Indian cohort, with SF3B3, PLRG1, and AQR being the most mutated splice factors (Table 8) across the samples (2/10) and the spliceosome complexes. Genes LSM4 and SF3B5 were the most upregulated across (7/10), whereas LSM14B, PRPF8, SRRM, and U2AF2 were the most downregulated in (4/10) samples (Figure 6A) in splice factor-specific context. We checked for the most spliced gene across subtypes and identified lncRNA GAS5 (SNHG2) and SNHG17, metalloprotease ADAM15, and core mRNA processing proteins RPS24 and UBA52 to be the most spliced. SNHG17 showed >27 alternatively spliced exons, particularly in high-grade samples, which was an exciting finding considering its non-coding function. In tumour samples, metalloprotease ADAM 15 showed exon usage, harbouring disordered regions with sites for Y715 and Y735 of the cytoplasmic tail that influence its association with its interacting partners in cellular signaling (Poghosyan et al., 2002). Further, we performed TSG and ONC analysis on the transcripts alternatively spliced (Supplementary Table S1) to find TSGs, PER1, and FAS and ONCs, BCL2L12, and MP2K2 were most alternatively spliced, might contribute to tumorigenesis.

We performed functional analysis to understand the consequences of alternative splicing events affecting domains, disordered regions, post-translational modifications (PTM), and active sites of proteins (Figure 6D). From earlier transcriptome analysis, the JAK-STAT pathway was enriched in the high-grade samples (Figure 5B); hence we wanted to check the alternatively spliced gene events associated with JAK-STAT signaling. We found alternative splice events related to the JAK-STAT pathway, particularly CD44 (Figure 6F), STAT1, FAS, and IRF1. There was an increase in differential exon usage of the variable region ex5-ex14 of the CD44 stems region, leading to its possible tumorigenic isoforms. Also, STAT1 and FAS harboured splice events that retained serine residues S701 and E705 (Figure 6G) in tumour samples with a dpsi value of −0.429 and *p*-value 3.617e-05 and S209 and T214 residues with dpsi value −0.727 with *p*-value 8.3539e-06, respectively, suggesting alterations in the post-translational event, phosphorylation, and change in the function of protein leading to loss of cell death activity. IRF1 showed a loss of DNA binding region, which might contribute to the dysregulated immune system and cell proliferation in Indian HGSOC. We found domains and PTM regions most affected in all samples and histotypes (Figure 6D). Among the genes affected at active sites by splice events were MUTYH, POLL, and UNG, particularly in POLL at K220, the site responsible for forming Schiff-base intermediate with DNA and UNG at D145, the proton acceptor site, indicative of the BER pathway being not functional to its full extent and with mutations harboured in the HR genes, seems fatal (Figure 6E).

Further, we performed pathway analysis (Table 9) of the AS events and found translation elongation common in all histotypes. On further analysis, genes revealed enrichment of ribosomal proteins involved in translational elongation. Out of 80 core ribosomal proteins, more than 50% were alternatively spliced across the samples. To check pathways altered other than those enriched by ribosomal proteins, we performed pathway analysis with alternatively spliced genes with absolute dpsi >0.2 and FDR <0.05 without ribosomal proteins to obtain pathways specific to the OVCa histotype.

**TABLE 9:** Enriched pathways in alternative splice events.

Sample histotype	Alternative splicing enriched pathways
ECys	Organelle Biogenesis and BER
SC	BER and Electron transport chain
LGSPC	mRNA splicing and Electron transport chain
HGSPC	Electron transport chain and TCA
MBOC	ETC, Organelle Biogenesis and DDR, Negative Regulators Of DDX58/IFIH1 Signalling
GCT	TCA and Glycosylation
HGSOC	Chromatin Modifying enzymes, Organelle biogenesis, TCA, ETC and mRNA splicing

The post-modifications were most affected by ASE events among OVCa histotypes. The BER pathway, POLL, and UNG in HGSOCs showed a loss of active sites essential for their repair function. Mutations in DDR and loss of BER functional genes may cause further genomic instability and result in a tumorigenic advantage.

Altered pathways, including Splicing, organelle biogenesis, energy metabolism, and Base excision repair, were explicitly in ASE events and not in the raw transcriptome.

To conclude, we plotted the number of genes impacted by mutation and splicing to find that significantly more genes are influenced by splicing than mutations (Figure 6H). This further highlights the importance of applying an integrated approach to omics studies.

## 4 Discussion

We have sequenced exome, transcriptome, and miRNA from 4 histotypes of ovarian cancer (12 samples; 10 tumours and 2 Normal) and analysed mutation, expression of genes, and its alternate spliced forms to obtain histotype and sample-specific alterations in pathways to unravel the mechanism of oncogenesis in Indian Cohort.

Mutation analysis showed HRD deficiency and samples with BRCA1 and BRCA2 mutations in 2/4 HGSOC samples and TP53 pathogenic mutations were reported (Supplementary Table S1). TP53 mutations were observed in 50% of the HGSOC samples unlike in the TCGA cohort where 96% of ovarian cancers were TP53 mutated (Network and The Cancer Genome Atlas Research Network, 2011). Likely pathogenic mutations were observed in RAD51B, the sensor and modulator of DNA damage, in serous cystadenoma. The highest tumour mutation burden was observed in HGSOC, followed by Granulosa cell tumour (GCT) with abundant MODERATE and HIGH impact variants. The samples with the highest mutation burden correlated with mutations in POLE in the Indian cohort as has been reported in other cohorts (Wang et al., 2018). Apart from the ones mentioned above, the most mutated cancer-implicated genes were ALK (7/10), PMS1 (4/10), and PTPRB (6/10).

Mucins MUC4 and MUC16 (CA125), MHC II transactivator CIITA along with transcriptional corepressor NCOR2 were the most mutated genes in about more than 8/10 samples in the Indian cohort. MUC4 mutation frequency observed in the Indian cohort was the same as seen in the TCGA cohort ∼80%. Mucins are over-expressed in multiple cancers and also modulate immune behavior, therefore can be targeted for therapy and not just prognosis as in the case of CA125 testing. Apart from downregulated NCOR2, MUC4, MUC16, and CIITA were overexpressed in HGSOC samples. Novel tumour-associated mutations in CIITA and MUC16 were recorded in the Indian cohort. Characterising these mutations leading to loss or gain of function might help understand the role of specific mutations in the Indian Cohort. In larger cohort studies of ovarian cancer transcriptomes showed NCOR2 as a biomarker for resistance (Fekete et al., 2020); lower expression observed in the Indian Cohort might be a good predictor of chemotherapy response.

Since the data on survival and remission after the treatment was lacking, conclusion could not be drawn.

WGCNA analysis identified modules and hub-specific genes specific to histotype samples. Serous cystadenoma showed module genes enriched in the collagen receptor signaling pathway. Previous studies have shown differential Procollagen I mRNA and protein detection in OVCa cells. The difference among the histotypes in the distribution of collagen I was also observed in cystadenomas, borderline tumours, and different grades of OVCa (Davidson, Trope, and Reich, 2014) (Zhu et al., 1995). Discoid Domain Receptor, DDR1 is expressed at high levels in OVCa and ST09, a curcumin derivative that blocks collagen receptor signaling (Ravindran et al., 2021), and can be used as a potential drug for HGSOCs. Similarly, in the endometrioid cyst, upregulation of Wnt signaling was observed and Granulosa cell tumours showed enrichment of antagonism of Activin and Follistatin in the Indian Cohort, of which a Phase I trial is carried out in Granulosa Cell Ovarian Cancer patients using an Activin inhibitor STM 434. In HGSOCs, TYROBP and SIRPG signaling was enriched, known to regulate NK cells and leukocyte adhesion, modulate immune cells, and play a role in TME. Whereas in the case of LGSPC, mRNA processing pathways were enriched to provide a selective advantage by promoting the transcription of erroneous mRNAs. HGSPC showed FOXP3 and RUNX1 mediated Treg signaling which was further seen in the immune profile plot as a high Treg fraction. The most downregulated TSG was PER1, in studies, PER1 expression levels correlated with infiltration neutrophils, regulatory T cells, and M2 macrophages (Chen et al., 2021). Immune infiltration was observed and was unique to each subtype and sample. Upregulated genes ERBB3 (HER3) and ERBB4 (HER) belong to the HER family of receptors and are implicated in breast cancer. Mucins are known to modulate the activity of ERRB. In the Indian cohort, along with the upregulation of ERBB3 and ERBB4, MUC4 was mutated across the samples.

From miRNA seq, miR-200 and miR-1269a families were highly expressed in HGSOC samples in the Indian cohort. Out of the six target genes regulated by miR-200 that matched TCGA OVCa expression, ZCCH24 expression correlated significantly with survival (Figure 4D). ZCCH24 is an RNA-binding protein that regulates mRNA splicing and has been implicated in immune cell infiltration in several cancers. The multidimensional analysis revealed the coordinated effect on the immune system and reasons for chemoresistance/sensitivity in the Indian cohort. Targeting singular targets might lead to cross-talks, causing resistance. Therefore combination therapy aiming different pathways might lead to better outcomes.

Mutations in splicing complexes were observed, namely, in SF3B3, PLRG1, and AQR. SF3B is a component of the U2 small nuclear ribonucleoprotein (snRNP), known to control AS in renal cancer and SF3B4 in ovarian cancer by regulating AS of RAD52 (Diao et al., 2022). On performing correlation studies, the number of splice events observed correlated with the upregulation of splice factors with R = 0.75 and pval = 0.031. In the splice events observed, most spliced genes were lncRNAs SNHG2 (GAS5) and SNHG17, considering its non-coding function was an interesting finding. Splice forms alter the oncogenic and tumour role of GAS5 in malignant tumours (Lin et al., 2022), and the role of non-coding RNA, SNHG17 in ovarian cancer needs to be investigated. ADAM15 splice events showed retention of residues Y715 and Y735 of the cytoplasmic tail, to facilitate motility and in turn proliferation. With consequential analysis to know impacted pathways and functional regions, translational elongation was most enriched across histotypes due to the presence of ribosomal proteins. Ribosomal proteins possess extra ribosomal functions to regulate cell growth, differentiation, immune signaling, DNA repair, and apoptosis apart from strongly. Intrinsically disordered regions (IDR) facilitate these interactions; hence ASE in core ribosomal proteins might impact their moonlighting role in the cellular environment. (Kang et al., 2021).

Apart from the mutation of JAK1, GSEA analysis revealed enrichment of JAK-STAT in HGSOC samples. Alternative splice events associated with the JAK-STAT pathway signify different players, particularly CD44, STAT1, FAS, and IRF1. CD44 showed an increase in exon usage to result in tumour associated forms and IRF1 with loss of DNA binding region. Whereas STAT1 and FAS showed retention of phosphorylated residue regions, this could cause a delay in cell death and prove beneficial for the tumour.

The post-modifications were most affected by ASE events among OVCa histotypes. The BER pathway, POLL, and UNG in HGSOCs showed a loss of active sites essential for their repair function. Mutations in DDR and loss of BER functional genes may cause further genomic instability and result in a tumorigenic advantage.

To conclude, the current study found population-specific and novel variants in tumour-specific contexts. Integrated exome and transcriptome revealed CIITA, MUC4, and MUC16 as possible drivers due to elevated expression and mutational hits in the Indian Cohort. From miRNAs, miR-200 was highly upregulated with its targets down in tumour samples, namely, ZCCHC24 with survival significance found. The most spliced gene was SNHG17 with >25 splice forms and tumour associated splice events of CD44, STAT1, FAS, and IRF1 further confirming the JAK-STAT pathway enrichment from GSEA. The limitation of the study is the sample number which requires further validation in a larger cohort. Finally, to summarise, integrated exome, transcriptome, and splicing patterns revealed different molecular signatures in the ovarian cancer histotypes of the Indian Cohort, implying the need for a multi-omics approach for prognostic and diagnostic purposes.

## Data Availability

The data presented in the study is deposited in the SRA repository, accession number PRJNA915025.

## Supplementary material

SUPPLEMENTARY FIGURE S1 Box plot showing raw library counts of all samples involved in the analysis on the left and on the right boxplot of voom normalised libraries.

SUPPLEMENTARY FIGURE S2 Blue tile plot showing clustering of sample on the basis of transcriptome of tumour and normal samples. Darker shades of blue indicate close relationship between samples.

SUPPLEMENTARY FIGURE S3 MA plots of OVCa histotype samples, showing differentially expressed genes highlighted in as blue dots.

SUPPLEMENTARY FIGURE S4 MA plots of OVCa histotype samples, showing differentially expressed genes highlighted in as blue dots.

SUPPLEMENTARY SHEET S1 Details of mutations present in candidate DDR, ONCs, and TSGs, Novel deleterious variants, Differential expressed ONCs and TSGs, most spliced genes across samples and Splice events in protein domains in samples.
