# Supplementary figures and images for: Multi-omics analysis of the Indian ovarian cancer cohort revealed histotype-specific mutation and gene expression patterns
Source: Front Genet. 2023 Apr 6;14:1102114. doi: 10.3389/fgene.2023.1102114 (PMC10117685; doi:10.3389/fgene.2023.1102114)

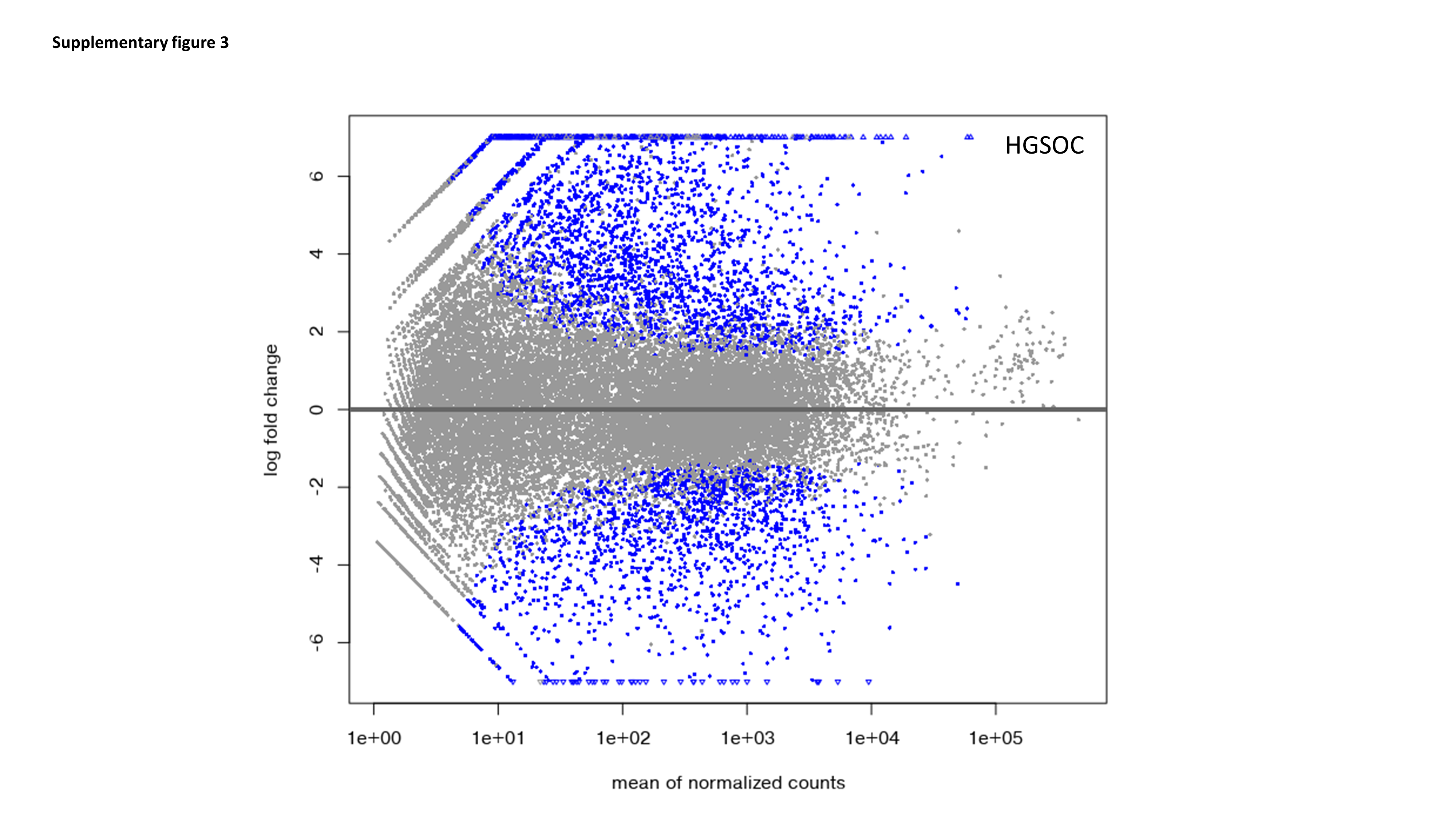

Supplement: Supplementary file 1 [file Image3.TIF]

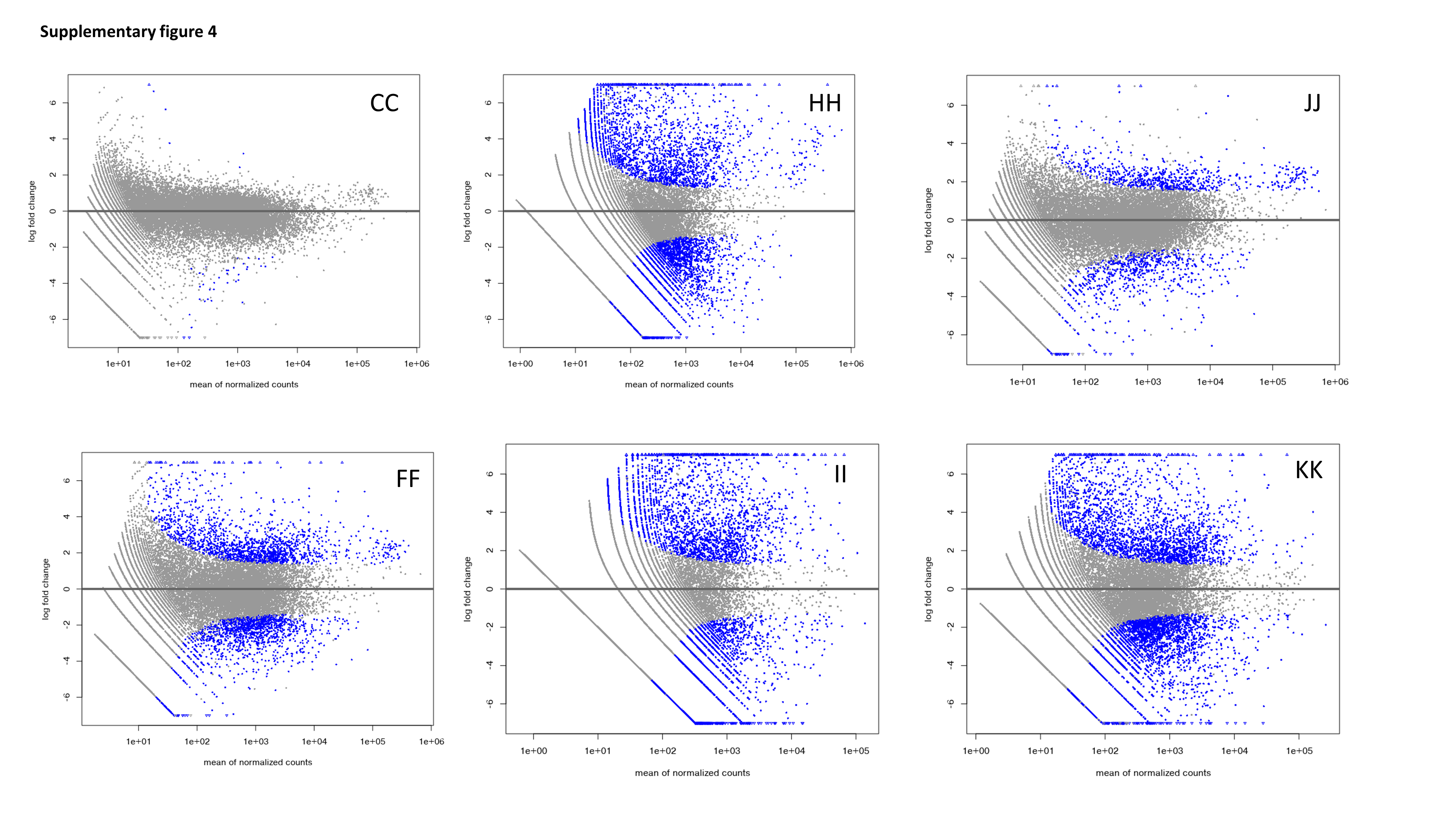

Supplement: Supplementary file 2 [file Image4.TIF]

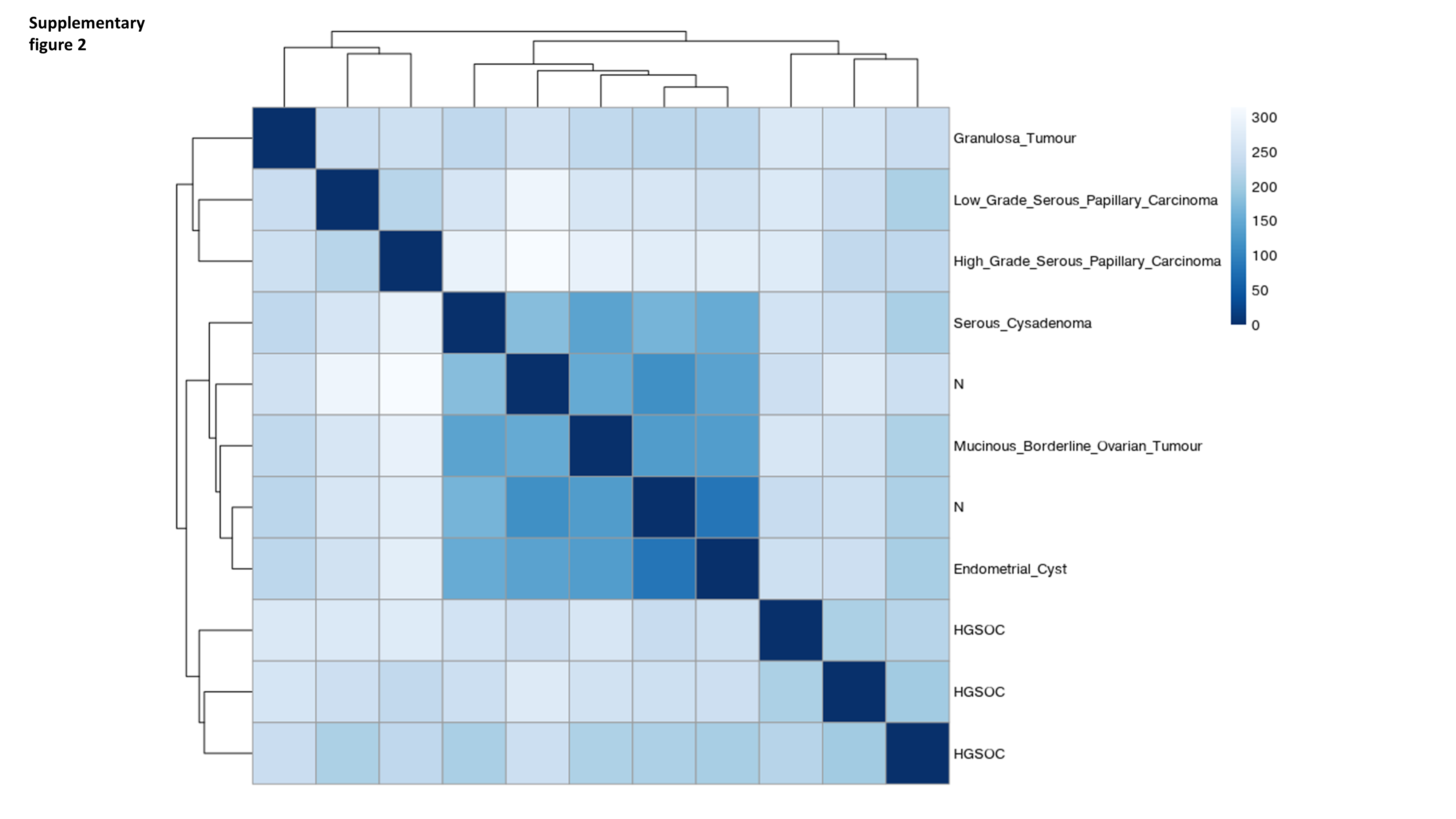

Supplement: Supplementary file 3 [file Image2.TIF]

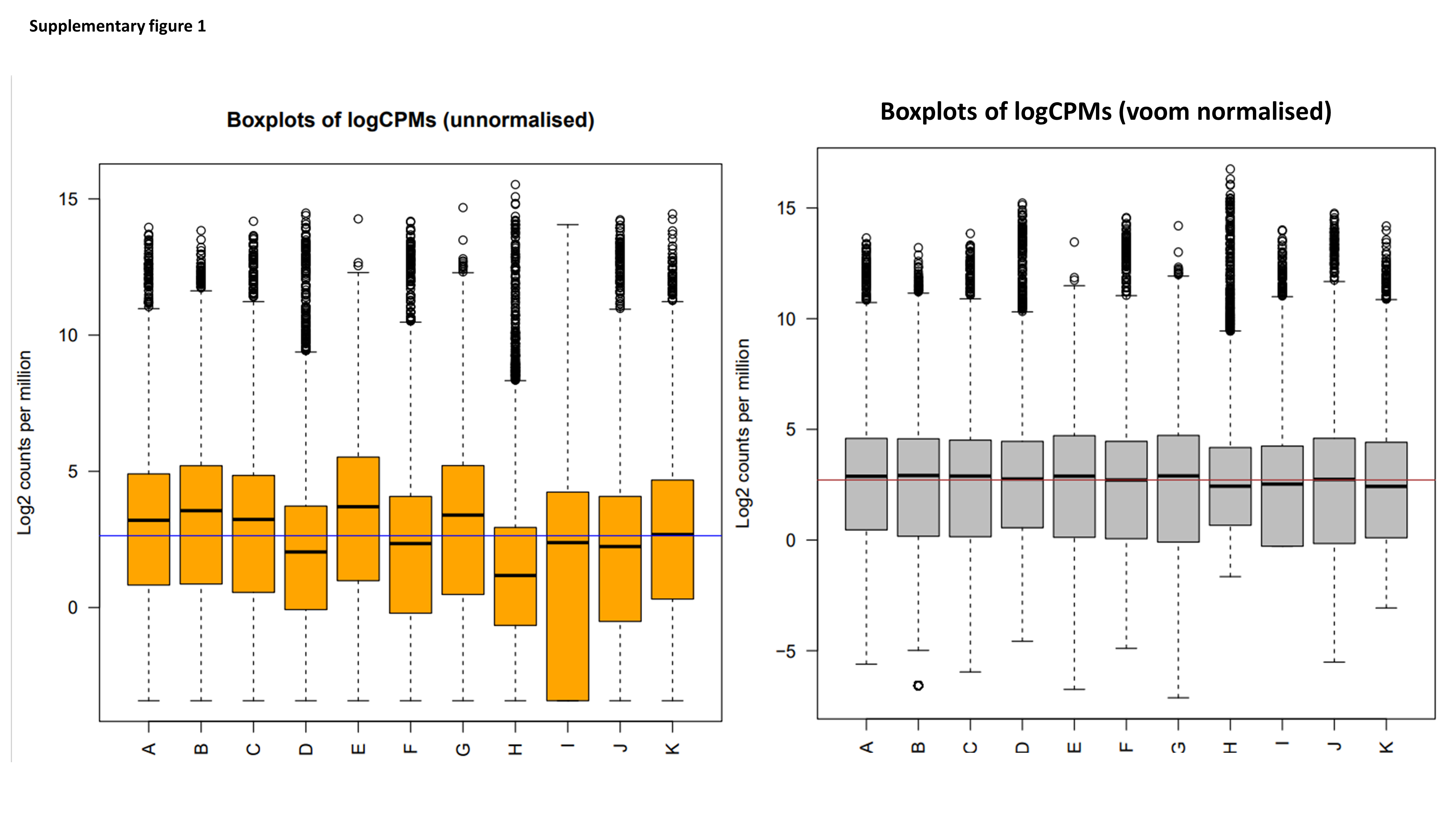

Supplement: Supplementary file 4 [file Image1.TIF]
